# Berberine Promotes Induction of Immunological Tolerance to an Allograft via Downregulating Memory CD8^+^ T-Cells Through Altering the Gut Microbiota

**DOI:** 10.3389/fimmu.2021.646831

**Published:** 2021-02-12

**Authors:** Feifei Qiu, Weihui Lu, Shulin Ye, Huazhen Liu, Qiaohuang Zeng, Haiding Huang, Chun-Ling Liang, Yuchao Chen, Fang Zheng, Qunfang Zhang, Chuan-Jian Lu, Zhenhua Dai

**Affiliations:** ^1^Section of Immunology and Joint Immunology Program, Guangdong Provincial Academy of Chinese Medical Sciences, Guangzhou, China; ^2^The Second Affiliated Hospital of Guangzhou University of Chinese Medicine, Guangzhou, China

**Keywords:** immunological tolerance, berberine, allograft rejection, memory T cell, gut microbiota

## Abstract

Emerging evidence has linked the gut microbiota dysbiosis to transplant rejection while memory T-cells pose a threat to long-term transplant survival. However, it's unclear if the gut microbiome alters the formation and function of alloreactive memory T-cells. Here we studied the effects of berberine, a narrow-spectrum antibiotic that is barely absorbed when orally administered, on the gut microbiota, memory T-cells, and allograft survival. In this study, C57BL/6 mice transplanted with islets or a heart from BALB/c mice were treated orally with berberine. Allograft survival was observed, while spleen, and lymph node T-cells from recipient mice were analyzed using a flow cytometer. High-throughput sequencing and qPCR were performed to analyze the gut microbiota. CD8^+^ T-cells from recipients were cultured with the bacteria to determine potential T-cell memory cross-reactivity to a specific pathogen. We found that berberine suppressed islet allograft rejection, reduced effector CD8^+^CD44^high^CD62L^low^ and central memory CD8^+^CD44^high^CD62L^high^ T-cells (T_CM_), altered the gut microbiota composition and specifically lowered *Bacillus cereus* abundance. Further, berberine promoted long-term islet allograft survival induced by conventional costimulatory blockade and induced cardiac allograft tolerance as well. Re-colonization of *B. cereus* upregulated CD8^+^ T_CM_ cells and reversed long-term islet allograft survival induced by berberine plus the conventional costimulatory blockade. Finally, alloantigen-experienced memory CD8^+^ T-cells from transplanted recipients rapidly responded to *B. cereus in vitro*. Thus, berberine prolonged allograft survival by repressing CD8^+^ T_CM_ through regulating the gut microbiota. We have provided the first evidence that donor-specific memory T-cell generation is linked to a specific microbe and uncovered a novel mechanism underlying the therapeutic effects of berberine. This study may be implicated for suppressing human transplant rejection since berberine is already used in clinic to treat intestinal infections.

## Introduction

The gut microbiota not only is involved in host physiological processes, including vitamin synthesis, metabolism and immune defense (1, 2), but also plays an important role in many diseases, including multiple sclerosis (3) and autoimmune arthritis (4). Recently, a growing number of studies revealed the relationship between the gut microbiota and allograft rejection and GVHD (5–13). Changes in urinary microbiota correlated with renal transplant outcomes (14, 15). It was shown that microbiota dysbiosis was obviously associated with post-operative complications, such as infection and graft rejection (16–18). Meanwhile, pretreatment of recipient mice with broad-spectrum antibiotics (ABX) also prolonged survival of minor antigen-mismatched skin transplants (19) while differences in gut microbes in mice derived from different vendors resulted in a different graft outcome (20). Acute infection could temporarily interrupt tolerance (21) while antibiotic pretreatment attenuated hepatic transplant injury (22). Although it remains not fully understood how microbiota regulates immunity, recent advances in the field showed that a short-chain fatty acid, butyrate, regulated energy metabolism and inflammation (23, 24). Interaction of microbiota and bile acids could also alter host metabolism (25). Furthermore, three major tryptophan metabolites that affect T cell function, including serotonin, kynurenine, and indole derivatives, were controlled by the gut microbiota (26). Thus, the gut microbiota could be a therapeutic target for preventing transplant rejection.

Berberine, a natural ingredient originally derived from the plant *goldthread*, has been widely used to treat bacteria-associated diarrhea as an antibiotic in China (27, 28). Berberine monomer is poorly absorbed by the gastrointestinal tract (29–32), and thus its modulation of gut microbiota has been hypothesized as one of the main mechanisms underlying its beneficial effects on diabetes (33). Moreover, some studies demonstrated that berberine ameliorated autoimmune diseases, including colitis, arthritis and experimental autoimmune encephalomyelitis (EAE), by inhibiting Th1 and/or Th17 responses in rodents (34–42). Nevertheless, the direct effects of berberine on transplant rejection remain undefined. This study was undertaken to investigate the effects of berberine on allograft rejection and its underlying mechanisms in a murine model. We found that berberine promoted long-term islet allograft survival and cardiac allograft tolerance induced by brief costimulatory blockade via shrinking alloreactive CD8+ T_CM_ pool through altering the gut microbiota. This study may be implicated for the treatment of transplant rejection in humans.

## Materials and Methods

### Animals

BALB/c and C57BL/6 male mice (6 to 8 weeks-old) were obtained from Guangdong Medical Laboratory Animal Center (Guangdong, China), while C3H/HeJ mice (6 to 8 weeks-old) were purchased from Jiangsu ALF Biotechnology company (Nanjing, China). Mice were bred and maintained under a specific pathogen-free condition. Animal protocols involved in this study were approved by the Animal Ethics Committee of Guangdong Provincial Academy of Chinese Medical Sciences.

### Treatment of Mice

Recipient mice were randomly divided into control groups and groups treated with berberine (Ber: 200 mg/kg body weight daily) or ABX via oral gavage. Both Ber (Alfa Biotechnology, China, purity >99%) and ABX (Solarbio, China) were prepared with sterilized water. Mice in ABX group received 200 μl of mixed antibiotics, including gentamycin (0.35 mg/ml), kanamycin (5.25 mg/ml), colistin (8,500 U), metronidazole (2.15 mg/ml), and vancomycin (0.5 mg/ml), as described previously (19). Mice were treated with Ber or ABX for 3 weeks after transplantation or until graft rejection, whichever came earlier. In some experiments, mice were pretreated with ABX for 6 days prior to transplantation and rested for 1 day before further treatment. To induce long-term allograft survival, some recipients received brief treatment with low-doses of anti-CD154 Ab (MR1) or CTLA4-Ig (Bio X Cell, West Lebanon, USA) at 0.2 mg on days 0 and 4.

### Murine Islet and Heart Transplantation

Islet transplantation was performed as described in our previous studies (43, 44). Briefly, 2 ml of collagenase V (1 mg/mL, Sigma) was injected into common bile duct of BALB/c donors. The pancreas was removed and incubated in a 37°C water bath for 16–18 min and the crude preparation was filtered through a 100-μm nylon cell-strainer. Islets were counted and ~400 islets were injected into the renal subcapsular space of C57BL/6 recipients. Recipient mice were rendered diabetic by a single injection of streptozotocin (180 mg/kg, Sigma) 10–14 days before transplantation. Primary graft function was characterized by a blood glucose level of <200 mg/dL for 48 h after transplantation. Graft rejection was defined as a rise in blood glucose to >300 mg/dL for 2 consecutive days after primary function. Cardiac donors were 7 to 8-week-old BALB/c (H-2^d^) mice while recipients were 7 to 8-week-old C57BL/6 mice (H-2^b^). Fully vascularized heterotopic heart transplantation was performed as described previously (45, 46).

### Flow Cytometry

Draining lymph node (dLN) and spleen cells were harvested and stained with anti-CD4-FITC, CD8-FITC, CD44-V450, CD62L-PE-Cy7, FoxP3-APC, EOMES-PE-Cy7, Bcl-6-PE, and anti-Thy1.1 (CD90.1)-PE Abs (eBioscience or BD Biosciences). To determine intracellular expression of FoxP3, EOMES, and Bcl-6, cells were fixed and permeated according to the protocol of Foxp3/Transcription Factor Fixation/Permeabilization Concentrate and Diluent Kit (eBioscience). Then, cells were stained with anti-FoxP3, EOMES, or Bcl-6 Abs and finally analyzed using FACSAria III (BD Biosciences). To purify CD8+ T_CM_ cells, cells were stained with anti-CD8-FITC, CD44-V450, and anti-CD62L-PE-Cy7 Abs and CD8+CD44^high^CD62L^high^ T cells were sorted out via FACSAria III (BD Biosciences). To purify CD3+ T cells, splenocytes were stained with anti-CD3-PE Ab and CD3+ cells then were sorted out.

### 16S rRNA Gene Amplification and Sequencing

Caecum microbiota from recipient mice were collected and used for DNA extraction. Then V4 region of 16S rRNA genes was amplified from purified DNA using specific PCR primers (F515/R806). Replicate PCR products were quantified and pooled. Finally, all determinant libraries were sequenced on an Illumina MiSeq platform.

### Sequencing Data Analysis

Sequencing data were analyzed via FLASH (Fast Length Adjustment of Short reads, v1.2.11) for generating Tags, which were subsequently clustered to OTU (Operational Taxonomic Unit) at 97% similarity by scripts of software USEARCH (v7.0.1090). Then OTU representative sequences were taxonomically classified using Ribosomal Database Project (RDP) Classifier v.2.2 (47) trained on the Greengenes database (48), utilizing 0.6 confidence value as cutoff.

### Bacteria Strains, Culture, and Preparation of Bacterial Suspension

*Bacteroides ovatus* (*Bacteroides ov*.) was purchased from ATCC (ATCC 8483), and cultured in anaerobically sterilized ATCC medium 260 containing tryptone, soytone, NaCl, and sheep blood (defibrinated) under strict anaerobic conditions overnight. *Bacillus ce*. was bought from BeNa Culture Collection (BNCC103930) and cultured in anaerobically sterilized medium 260. Briefly, cultures were centrifuged at 9,000 rpm for 10 min and re-suspended in PBS containing 10% glycerol to 5 × 10^10^ colony-forming units (CFUs)/ml.

### Quantification of the Abundance of Bacteria Via qPCR

Bacterial relative abundance was determined by qPCR using the ABI ViiA 7 detection system (Thermo Fisher Scientific) and THUNDERBIRD SYBR qPCR Mix (TOYOBO). The relative quantity of each bacteria was calculated by the ΔCt method and was normalized to the amount of total bacteria (16S). The primers for 16S rRNA gene were used as the following: Forward, 5′-ACTCCTACGGGAGGCAGCAG-3′, Reverse, 5′-ATTACCGCGGCTGCTGG-3′. For taxa assays, the following primers were used: *Bacteroides ov*., Forward, 5′-AAGTCGAGGGGCAGCATTTT-3′, Reverse, 5′- CACAACTGACTTAACAATCC-3′; and *Bacillus ce*., Forward, 5′-TTCAAATTCAAAAGAATGTTGAAGAAGG-3′, Reverse, 5′-GATTTGTTTGCTTATTCATTTCATCAC-3′.

### Analysis of T Cell Apoptosis and Proliferation *in vitro*

FACS-sorted CD3+ T cells derived from Thy1.1+ C57BL/6 mice were sorted out, labeled with CFSE and then cultured with BALB/c splenocytes in the presence of berberine or CsA. Briefly, cells were stained for Annexin V-APC according to the protocol of Annexin V Apoptosis Detection Kit (BD Biosciences). For proliferation assays, cells were labeled with 1 μM CFSE (Invitrogen, Germany) at room temperature for 15 min. Then, cells (2 × 10^5^ cells/well) were cultured with mitomycin C-treated BALB/c splenocytes (4 × 10^5^ /well) in 96-well plates in complete RPMI-1640 medium in the presence of IL-2 (10 ng/ml, Peprotech) at 37°C for 4 days. These cells were treated with berberine (0.1 and 0.5μM) or CsA (100 mM). To measure memory T-cell response to *Bacillus ce*., CD8+ T-cells from previously transplanted mice were purified, labeled with CFSE and cultured with self-APCs in the presence of inactivated *Bacillus ce*. (1 × 10^5^ CFU/well), for 24 h. *Bacillus ce*. was inactivated by incubating at 65°C for 1 h (49), while self-APCs (50) were obtained from C57BL/6 splenocytes depleted of CD3^+^ T cells using magnetic micro-beads. Finally, cells were harvested and their proliferation was measured after gating on Thy1.1^+^ populations using FACS.

### Measurement of IFN-γ and IL-17A via ELISA

The protein levels of IFN-γ and IL-17A were measured using ELISA kits according to the manufacturer's instructions (Boster, China), and the absorbance was read at 450 nm in a microplate spectrophotometer (Thermo Fisher Scientific, USA).

### Statistical Analysis

Comparisons of the means were performed using one-way ANOVA or Student *t*-test. Data analyzed using GraphPad Prism 7 (GraphPad Software, La Jolla, CA, USA). The analysis of graft survival was performed using Kaplan–Meier method (log-rank test). A value of *P* < 0.05 was considered statistically significant.

## Results

### Berberine Inhibits Murine Islet Allograft Rejection

To investigate the effects of berberine on allograft survival, C57BL/6 mice were transplanted with islets from BALB/c mice and treated with berberine (Ber) or broad-spectrum antibiotics (ABX). As shown in Table 1, berberine extended islet allograft survival in immune competent wild-type mice compared to the control, with a statistical significance (median survival time or MST = 33 vs. 14 days, *P* < 0.05), while ABX did not (MST = 18 vs. 14, *P* > 0.05), suggesting that berberine can regulate alloimmunity.

**Table 1:** Berberine suppresses murine islet allograft rejection.

**Group**	**Survival time**	**Median survival**
	**(days)**	**time (MST)**
Control	11,12,12,13,14,14,15,16,17	14
ABX	13,14,15,17,19,22,24,25	18
Berberine	21,23,27,31,35,36,38,41	33*

### Berberine Diminishes Effector and Memory CD8^+^ T Cells

**Figure 1:**
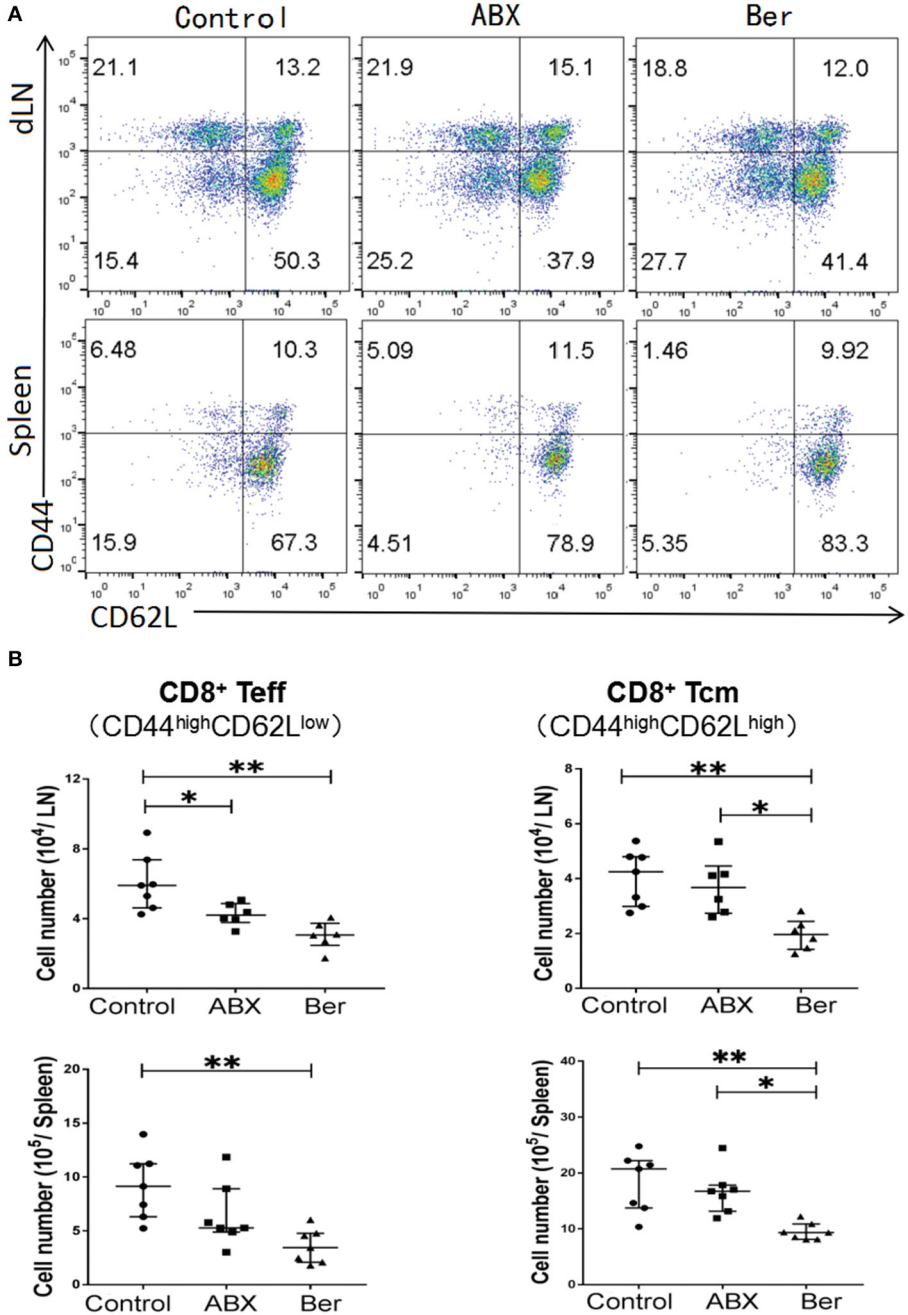
Berberine reduces effector and memory CD8+ T cells. Cells from dLN and spleen of recipient mice were harvested 4 weeks after islet transplantation and treatment with berberine. The percentages of CD8^+^CD44^high^CD62L^low^ (T_eff_) and CD8^+^CD44^high^CD62L^high^ (T_cm_) cells were measured via a flow cytometer **(A)**, while their absolute numbers were also calculated **(B)**. Data of individual values (b) are presented as median ± interquartile range (*P < 0.05 and **P < 0.01, *n* = 6–7 mice/group).

To determine whether berberine would regulate effector CD8^+^ T cells (CD44^high^CD62L^low^, T_eff_) and central memory CD8^+^ T cells (CD44^high^CD62L^high^, T_CM_), dLN and spleen cells from recipient mice treated with berberine or ABX were isolated 4 weeks after transplantation. As shown in Figures 1A,B, either berberine or ABX decreased CD8^+^ T_eff_ number in dLNs while berberine, but not ABX, also reduced their number even in the spleen. However, it was berberine but not ABX that lowered CD8^+^ T_CM_ number in dLNs and spleen of recipient mice (Figure 1B). Interestingly, berberine did not significantly alter CD4+FoxP3+ Tregs (Supplementary Figure 1).

Four weeks after transplantation, berberine also significantly reduced CD8+ T_CM_ cell number in naïve mice without transplantation (Supplementary Figure 2). Moreover, we tracked CD8^+^/CD4^+^ Thy1.1^+^ T_CM_ cell formation in transplanted mice adoptively transferred with naïve Thy1.1^+^ T cells. CD44^high^CD62L^high^CD8^+^/CD4^+^T_CM_ numbers within Thy1.1^+^ population were determined via FACS. We found that berberine, but not ABX, reduced the number of Thy1.1^+^CD8^+^, but not Thy1.1^+^CD4^+^, T_CM_ cells in both dLNs and spleen (Supplementary Figure 3), indicating that it downregulates CD8^+^, but not CD4^+^, T-cell memory. To further track alloreactive CD8+ T_CM_ cells that are still non-artificial or non-transgenic, CD3+ T cells isolated from naïve Thy1.1+ B6 mice were stained with CFSE and stimulated with BALB/c splenocytes in an MLR for 4 days. Thy1.1+CD8+ T cells that underwent at least two divisions were sorted and injected into C57BL/6 mice that were then transplanted with BALB/c islets and treated with berberine. We found that alloreactive Thy1.1+CD8+ T_CM_ cell number was decreased by berberine, but not ABX (Supplementary Figure 4). Finally, berberine did not promote the apoptosis of CD4^+^ T, CD8^+^ T, CD19^+^, and CD11c^+^ cells (Supplementary Figure 5), suggesting that orally administered berberine is non-cytotoxic.

### Berberine Suppresses EOMES Expression in CD8^+^ T Cells *in vivo*

**Figure 2:**
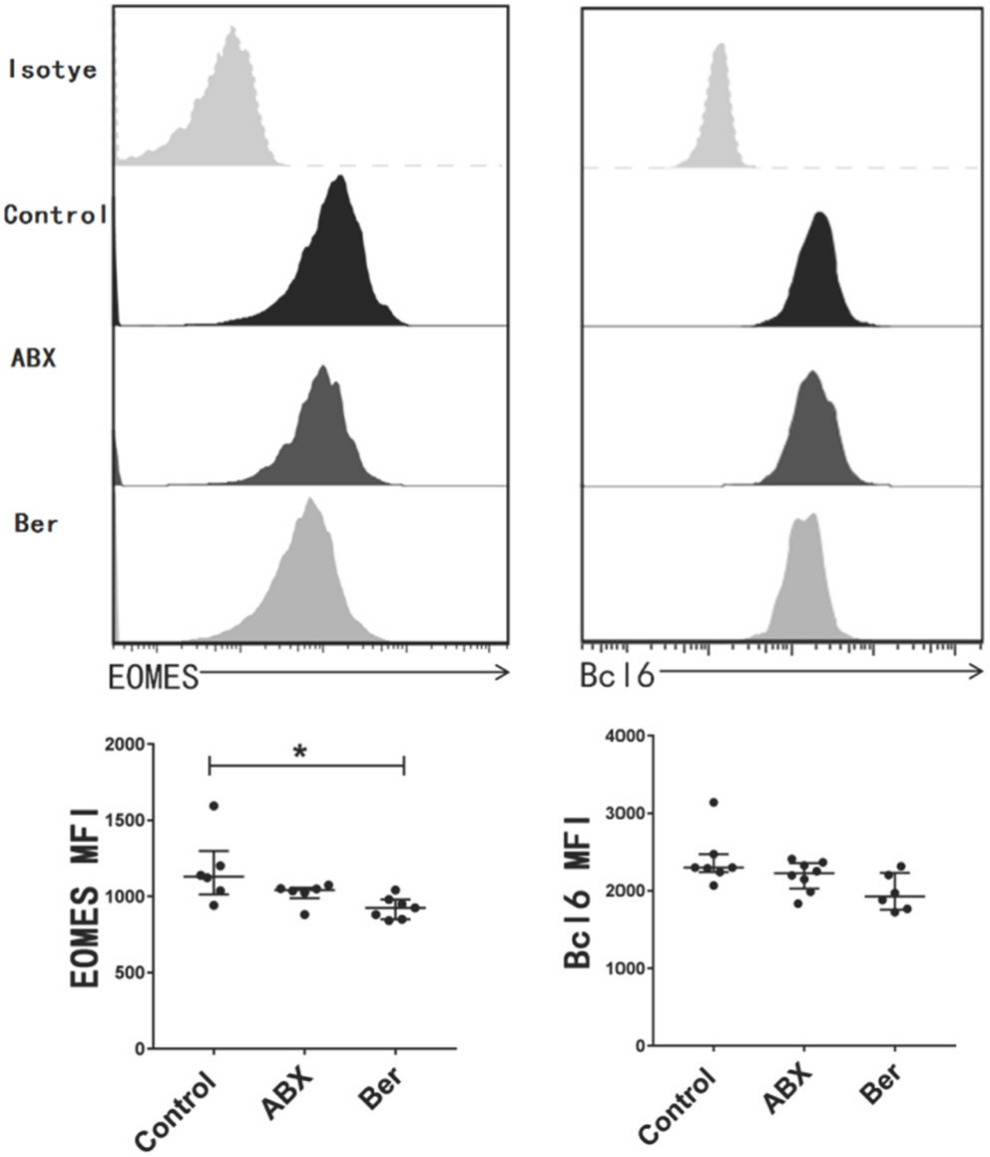
Berberine inhibits the expression of EOMES in CD8+ T cells. dLN cells were harvested 14 days after allotransplantation with various treatments. The expression of EOMES and Bcl-6 in CD8+ T cells was detected via a flow cytometer and quantified as mean fluorescence intensity (MFI). Histogram is shown after gating on CD8+ population. Plots of individual values are presented as median ± interquartile range (*P < 0.05, *n* = 6–8 mice/group).

EOMES, a transcription factor, is considered to be crucial for the maintenance of T_CM_ cells (51, 52). Bcl-6 is another transcription factor that is also important for the generation of T_CM_ cells (53). We found that berberine downregulated EOMES expression in CD8+ T cells from recipient mice, but did not significantly reduce Bcl-6 expression (Figure 2). However, there was no significant difference in their expression between ABX and control groups.

### Berberine Alters the Composition of the Gut Microbiota in Recipient Mice

**Figure 3:**
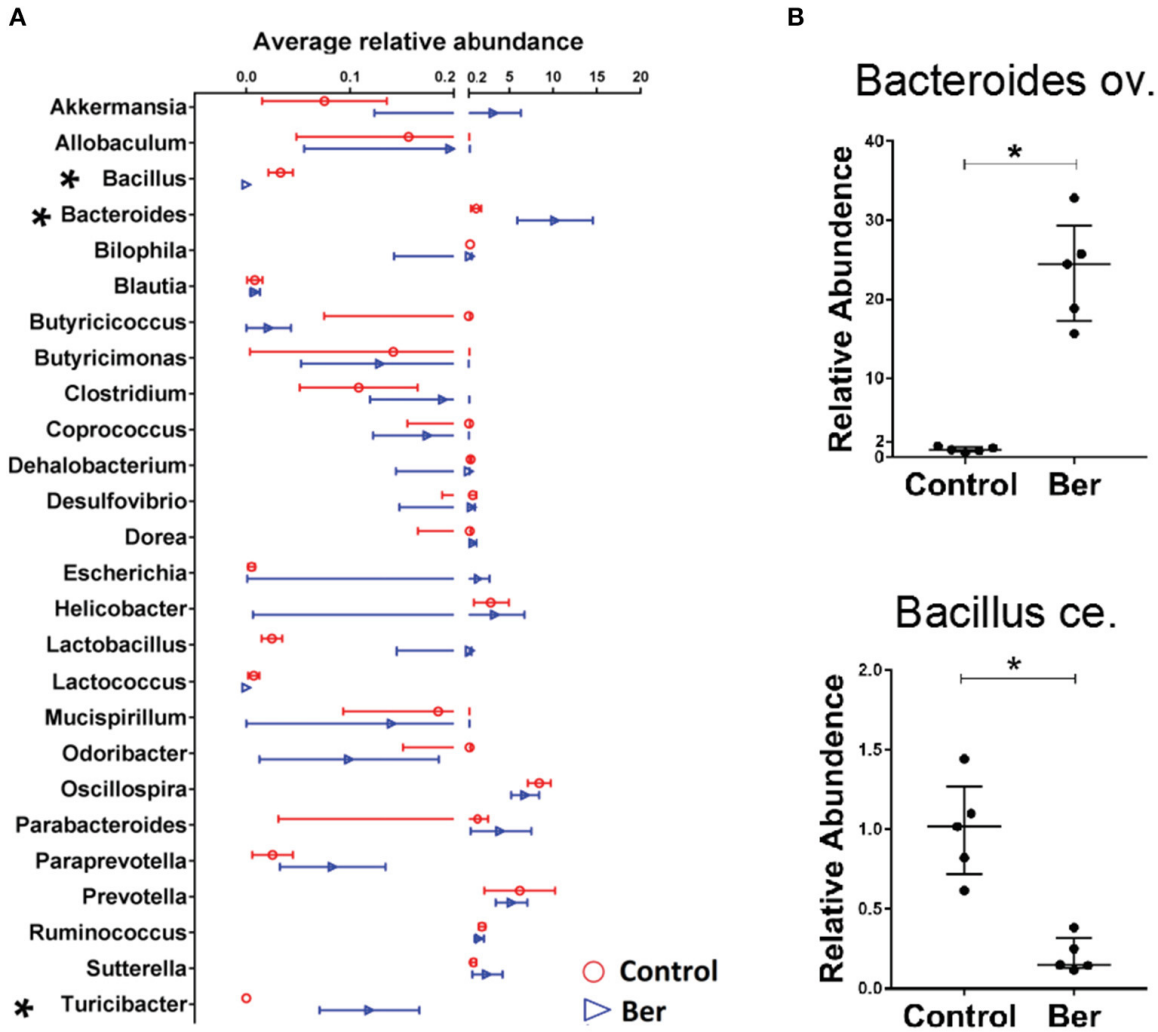
Berberine alters the composition of gut microbiota in recipient mice. Bacterial DNA was isolated from the cecum of B6 recipient mice transplanted with BALB/c islets 14 days after berberine treatment, and was analyzed via high-throughput sequencing. **(A)** The distribution of the top 26 bacterial taxa in berberine-treated and control groups at genus level (*n* = 4/group, shown as mean ± SEM, *P < 0.05). **(B)** Quantitative PCR was performed for validation of relative abundance of *Bacteroides ov*. and *Bacillus ce*. in caecum of control and berberine-treated mice (*n* = 5 mice/group, shown as median ± interquartile range with control group being set as 1.0).

To characterize the potential changes in gut flora 14 days after transplantation, bacterial DNA was extracted from caecal samples of recipients and 16S rRNA sequencing was performed. Berberine did not significantly decrease overall bacterial richness, as determined by analysis of OTUs (operational taxonomic units), which contrasted with a reduction in bacterial richness in ABX-treated mice (data not shown). Therefore, berberine treatment did not alter general bacterial richness. Furthermore, we analyzed the microbial abundance to identify berberine-sensitive microorganism. Among the top 26 genus-level taxa, berberine-treated recipients had much lower relative abundance of *Bacillus* in their microbiota and higher abundance of *Bacteroides* and *Turicibacter* (Figure 3A). Quantitative PCR also was performed to validate the sequencing results, and the expansion of *Bacteroides ov*. and contraction of *Bacillus ce*. were indeed observed in the cecum microbiota of berberine-treated mice (Figure 3B).

### Berberine Promotes Long-Term Islet Allograft Survival Under the Cover of Costimulatory Blockade While Addition of Exogenous CD8+ T_CM_ Cells or *Bacillus ce*. Reverses It

**Figure 4:**
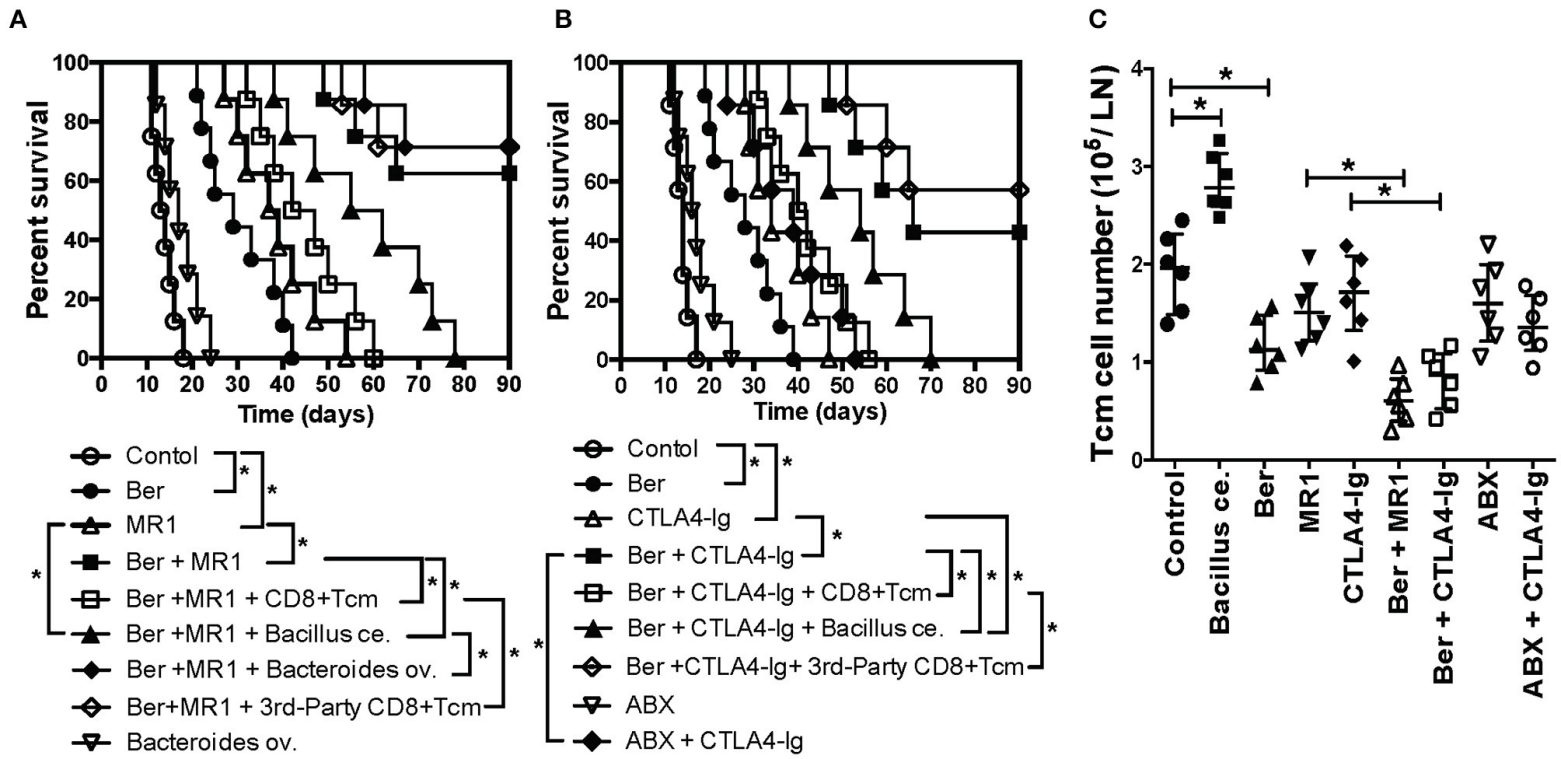
Berberine promotes long-term islet allograft survival induced by costimulatory blockade while addition of exogenous CD8+ T_CM_ cells or *Bacillus ce*. reverses the long-term islet survival. C57BL/6 mice were transplanted with islets from BALB/c mice and treated with berberine for 3 weeks and/or MR1/CTLA4-Ig on days 0 and 4. On day 28, some of transplanted mice that were treated with berberine plus MR1 **(A)** or plus CTLA4-Ig **(B)** received a single dose of bacterial strain (*Bacillus ce*. or *Bacteroides ov*.: 1 × 10^7^ CFU) or 1 × 10^6^ CD8+ T_CM_ cells isolated from an untreated B6 recipient that was previously transplanted/primed with BALB/c or C3H/HeJ skin (3rd-Party). Islet allograft survival was observed (*n* = 7–9 mice, *P < 0.05). In some groups, transplanted mice were also treated with ABX and/or CTLA4-Ig. **(C)** Draining dLN cells were harvested and assessed using a flow cytometer 4 weeks after transplantation with various treatments. The absolute cell numbers of CD8^+^CD44^high^CD62L^high^ (T_CM_) cells were measured via FACS. Data are presented as median ± interquartile range (*P < 0.05, *n* = 6 mice/group).

Since berberine reduced CD8+ T_CM_ formation, we then asked if berberine would help induce long-term islet allograft survival. We found that treatment with berberine plus either anti-CD154 Ab (MR1) (Figure 4A) or CTLA4-Ig (Figure 4B) induced long-term islet allograft survival (>90 days) in some recipients although berberine, MR1 or CTLA4-Ig alone extended islet allograft survival. On day 28, some groups of transplanted mice that were treated with berberine plus MR1 or CTLA4-Ig also received a single dose of bacterial strain (*Bacillus ce*. or *Bacteroides ov*.: 1 × 10^7^ CFU) or CD8+ T_CM_ cells derived from C57BL/6 recipients that were previously transplanted/primed with either BALB/c or C3H/HeJ skin (3rd-Party). As shown in Figure 4, administration of alloreactive, but not 3rd -party, CD8+ T_CM_ cells reversed the long-term islet allograft survival, with all recipients rejecting their allografts within 60 days, and so did *Bacillus ce*. transplantation. However, addition of *Bacteroides ov*. failed to alter long-term islet survival induced by Ber + MR1 (Figure 4A), while treatment with ABX + CTLA4-Ig also did not further extend the islet allograft survival compared to CTLA4-Ig alone (Figure 4B). Thus, although berberine increased the abundance of *Bacteroides ov*., the latter did not prolong allograft survival.

### *Bacillus ce*. Transplantation, but Not Costimulatory Blockade, Increases CD8^+^ T_CM_ Cells

To examine direct effects of *Bacillus ce*. on CD8+ T_CM_ generation, we performed re-colonization of *Bacillus ce*. in some islet recipients. CD8+ T_CM_ cells were quantified via FACS analyses. As shown in Figure 4C, *Bacillus ce*. colonization increased CD8+ T_CM_ cells. However, colonization of *Bacteroides ov*. did not alter their number (data not shown). Interestingly, either ABX or costimulatory blockade alone failed to significantly change their number, whereas treatment with Ber + MR1 or Ber + CTLA4-Ig further reduced their number compared to MR1 or CTLA4-Ig alone (Figure 4C). These results suggest that *Bacillus ce*. upregulates CD8+ T_CM_ cells while berberine does the opposite, with the costimulatory blockade of CD154 or CD28 alone failing to alter CD8+ T_CM_ formation.

### Berberine Induces Cardiac Allograft Tolerance Under the Cover of Costimulatory Blockade

**Figure 5:**
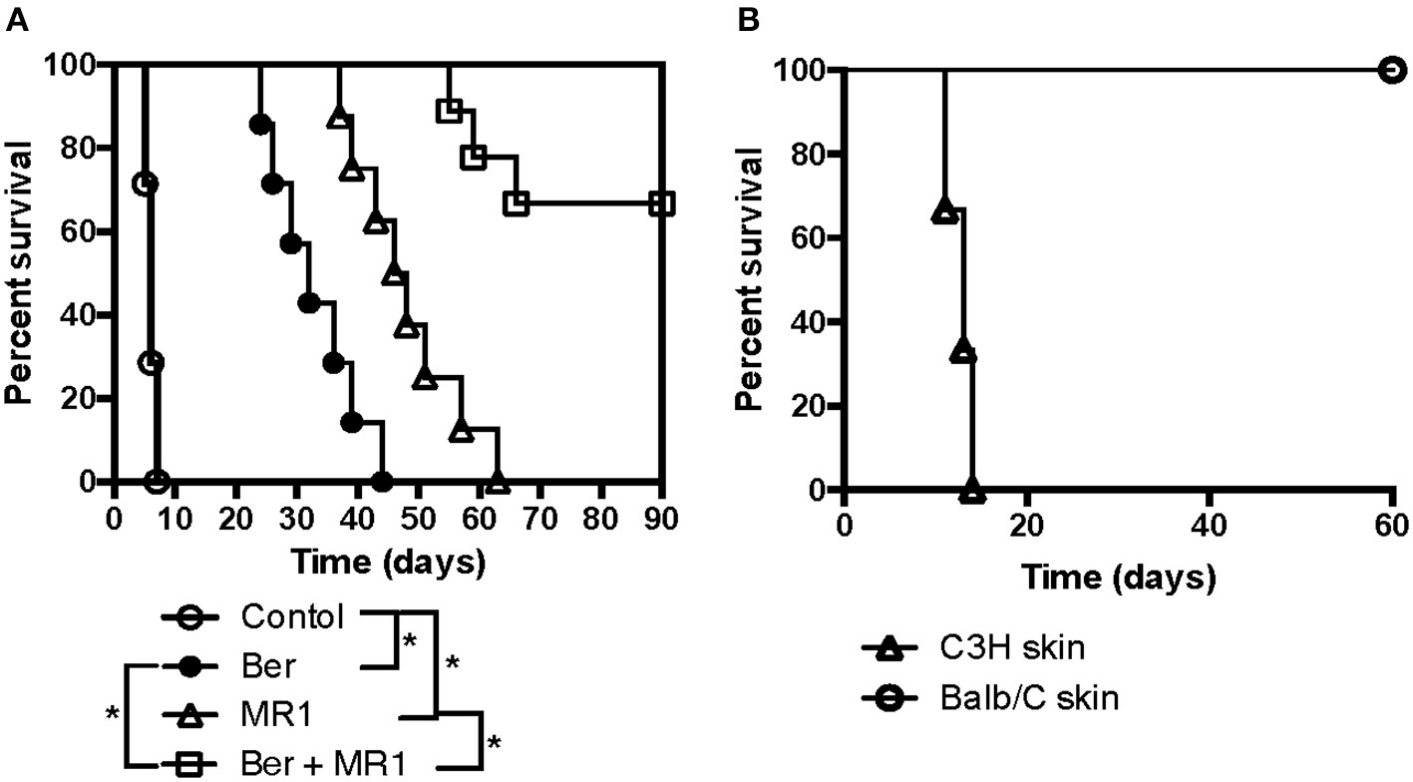
Berberine induces cardiac allograft tolerance under the cover of costimulatory blockade. To further confirm if berberine would help induce long-term allograft survival or tolerance in solid organ transplantation, C57BL/6 mice received a heart derived from a BALB/c mouse and were treated with berberine and/or MR1. Cardiac allograft rejection was observed (*n* = 7–9 mice/group, *p < 0.05) **(A)**. Moreover, C57BL/6 recipients with an accepted cardiac allograft (BALB/c) for 90 days were transplanted with a skin graft from a BALB/c or C3H/HeJ (third-party) donor (*n* = 3 mice/group), and skin allograft rejection was observed **(B)**.

To further confirm if berberine would help induce long-term allograft survival or tolerance in solid organ transplantation, C57BL/6 mice received a heart derived from BALB/c mice and were treated with berberine and/or MR1. As shown in Figure 5A, we found that berberine alone indeed delayed cardiac allograft rejection (MST = 32 vs. 6 days) while berberine plus MR1 induced long-term cardiac allograft survival (>90 days) in 6 of 9 recipients. Among the 6 recipients with an accepted cardiac allograft, 3 of them rejected a new skin allograft from C3H/HeJ donor while the rest of the recipients accepted a new one from BALB/c donor (Figure 5B), suggesting that berberine induces allospecific cardiac transplant tolerance.

### Berberine, at Low Concentrations Close to Those Achieved in Blood *in vivo*, Does Not Suppress T Cell Proliferation and Differentiation *in vitro*

**Figure 6:**
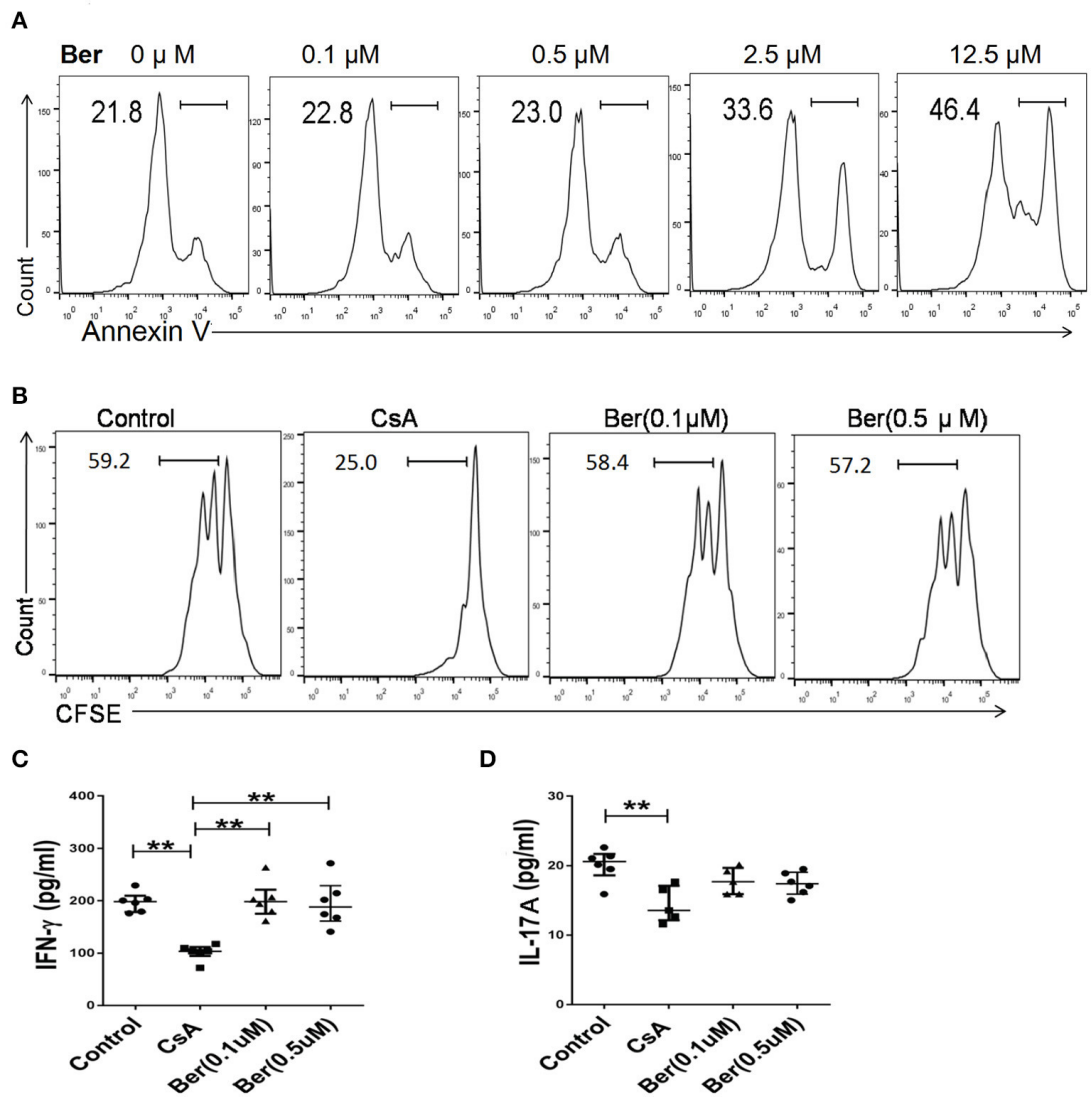
Berberine, at maximal concentrations achieved in the blood upon oral administration, does not inhibit T cell response *in vitro*. **(A)** FACS-sorted CD3+ T cells derived from Thy1.1+ C57BL/6 mice were sorted out and cultured with mitomycin-C-treated BALB/c splenocytes in the presence of various concentrations of berberine or CsA for 4 days, as detailed in the supporting information (item 2.3). Cells were then stained with Annexin V to detect their apoptosis. **(B)** CD3+ T cells, as described above, were labeled with CFSE before their culture. Four days later, they were stained for surface Thy1.1 and analyzed via FACS. Histograms were gated on Thy1.1+ T cells and one of the three sets was shown. Moreover, the culture medium was collected to determine the level of IFN-γ **(C)** and IL-17A **(D)** via ELISA. Plots of individual values are presented as median ± interquartile range (*P < 0.05, **P < 0.01, *n* = 5–6 mice/group from three separate experiments).

Orally administered berberine is barely absorbed in the intestinal tract, with a maximal concentration of <0.1 μM achieved in the blood (29–32). To determine if berberine directly inhibits T cell activation *in vitro*, FACS-sorted CD3+ T cells from Thy1.1+ C57BL/6 mice were stimulated with mitomycin C-treated BALB/c splenocytes for 4 days. As shown in Figure 6A, T cell apoptosis started to increase when berberine was used at 2.5 μM and reached 46.4% when used at 12.5 μM. Importantly, berberine, at either 0.1 or 0.5 μM, which did not yet promote T cell death, did not suppress T cell proliferation whereas CsA did so (Figure 6B). Similarly, berberine failed to reduce IFNγ (Figure 6C) or IL-17A (Figure 6D) level in the supernatant. These findings indicate that berberine, at relatively low concentrations that can be maximally achieved in the blood following oral administration, does not inhibit T cell response in an MLR, a setting of alloreactivity.

### CD8+ T Cells From Mice Previously Primed With BALB/c Skin Rapidly Respond to *Bacillus ce. in vitro*

**Figure 7:**
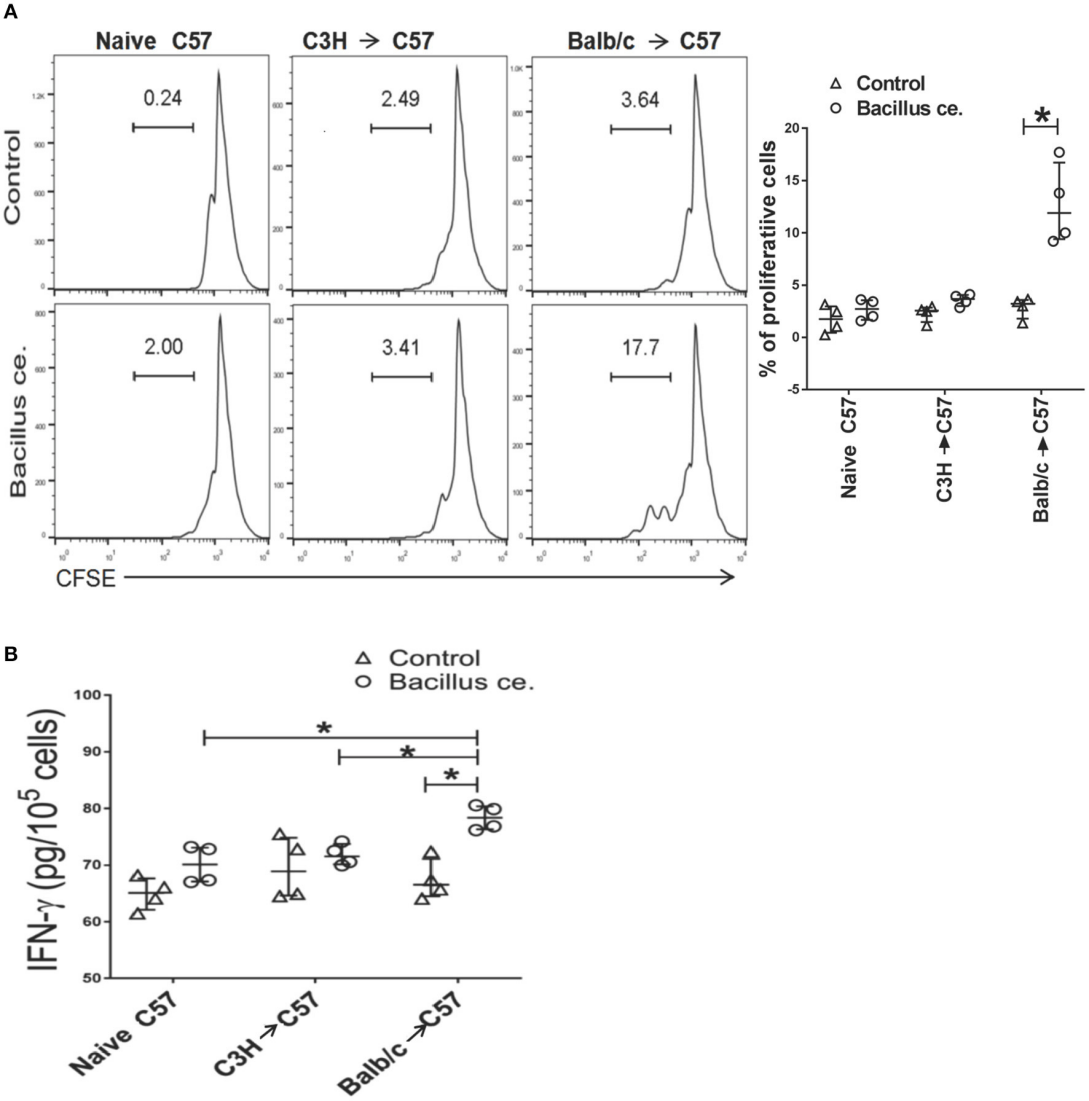
*Bacillus ce*. induces *in vitro* proliferation and IFN-γ expression by CD8^+^ T cells derived from recipient mice that were primed with BALB/c skin. FACS-sorted CD8+ T cells derived from naïve or C57BL/6 recipients transplanted with BALB/c or C3H/HeJ skin 4 weeks earlier were stained with CFSE and cultured in the presence of inactivated *Bacillus ce*. plus inactivated self APCs obtained from C57BL/6 splenocytes depleted of CD3+ T cells via magnetic micro-beads detachment. Twenty-four hours later, CD8+ T cell division was measured via FACS analysis after gating on CD8+ population **(A)**, while one representative of three separate sets of histograms is shown. Besides, IFNγ expression by the lyzed cells was also measured *via* ELISA **(B)**. Data of individual values are presented as median ± interquartile range (*P < 0.05, *n* = 4 mice/group).

To determine whether alloreactive memory CD8+ T cells developed in transplanted mice would rapidly cross-react to *Bacillus ce*., FACS-sorted CD8+ T cells derived from C57BL/6 recipients previously transplanted with BALB/c or C3H/HeJ (third-party) skin were stained with CFSE and cultured with inactivated *Bacillus ce*. and self APCs. CD8+ T cell division was measured via FACS while IFNγ expression was measured using ELISA. As shown in Figure 7A, 17.7% of CD8+ T cells, which were derived from C57BL/6 recipients previously transplanted with BALB/c skin, proliferated compared with only 2% of the cells from untransplanted naïve mice or 3.6% of the cells from those transplanted with skin of C3H/HeJ mice. Few control cells without *Bacillus ce*. stimulation proliferated. Furthermore, CD8+ T cells derived from C57BL/6 recipients previously transplanted with BALB/c skin also produced more IFNγ than control cells or those from recipients previously transplanted with C3H/HeJ skin (a third-party) (Figure 7B). These findings indicate that CD8+ T cells from transplanted/primed mice contain BALB/c-specific memory T cells rapidly cross-reacting to *Bacillus ce*, but do not necessarily imply that this cross-reactivity is limited to BALB/c donor antigens.

## Discussion

Using murine models of islet and cardiac allotransplantation, we found that berberine, a natural compound used to treat bacteria-associated diarrhea, delayed acute allograft rejection, and induced long-term allograft survival or tolerance under the cover of brief costimulatory blockade. In contrast, broad-spectrum ABX alone did not significantly delay acute rejection of a fully MHC-mismatched allograft, which is consistent with a previous study (54). Instead, Lei et al. demonstrated that ABX alone mainly extended minor antigen-mismatched skin graft survival (19). Collectively, previous studies and ours indicated that ABX did not dramatically suppress fully MHC-mismatched allograft rejection. Further, we found that berberine repressed CD8+ T_CM_ without a confounding effect on CD4+Foxp3+ Tregs. In contrast, ABX resulted in a reduction in Treg frequency in recipient mice, which was consistent with a recent study by Guo et al. (12). This phenomenon may have restricted otherwise beneficiary effects of ABX on allograft survival.

Previous studies have shown that berberine attenuates autoimmunity in animal models by inhibiting Th1/Th2 responses (34–42). For those studies *in vitro*, berberine was used at a concentration of ≥5 μM. However, it's well-known that the concentrations of berberine can not reach more than 0.1 μM upon the oral administration because it is barely absorbed by the intestine (29–32). We found that berberine, at a concentration of up to 0.5 μM, did not inhibit T cell proliferation and Th1/Th17 responses in an MLR, an *in vitro* setting of alloimmunity, indicating that berberine in our model does not directly suppress alloreactive Th1/Th17 responses. In contrast, we demonstrated that a higher concentration of berberine, at 12.5 μM, caused significant T cell apoptosis, indicating its cytotoxicity *in vivo* if it is directly injected. Moreover, previously studies were concerning animal models of autoimmunity, but not alloimmunity. It is ruled out that berberine, even with ultra-low concentrations in the blood, may still moderately suppress autoimmunity, directly or through altering the gut microbiota *in vivo*. At higher concentrations, such as 50–100 μM, berberine might also have suppressed Th1/Th17 cells by killing T cells in those studies. Therefore, it's very likely that berberine helps promote long-term allograft survival induced by costimulatory blockade via indirectly altering the gut microbiota, instead of directly inhibiting Th1/Th17 responses, given that berberine simply could not reach 0.1 μM in the blood after oral administration (29–32) and that, at up to 0.5 μM, it still did not suppress alloreactive T cell responses in our study *in vitro*. Importantly, our findings that treatment with berberine plus ABX failed to suppress allograft rejection also indicate that berberine does not directly inhibit alloreactive T cells in the presence of ABX.

Donor-specific memory T cells pose a significant challenge to long-term allograft survival. The presence of memory T cells in the blood of kidney graft recipients has been associated with poor outcomes in clinical transplantation (55, 56). Studies in animal models also revealed that memory T cells triggered rapid allograft rejection (57–59). Moreover, endogenous memory CD8^+^ T cells could still respond to alloantigens despite costimulatory blockade of CD28-CD80/86 or CD40-CD154 (60) while CD122 as a component of high-affinity IL-15 receptor was critical for memory CD8+ T cell recall response that was also independent of CD28 signaling (61). Our study revealed that berberine downregulated both CD8+ T_eff_ and CD8+ T_CM_ cells, implying that it is a potential agent to be used for induction of transplant tolerance. Interestingly, a previous study demonstrated that costimulatory blockade-mediated lung allograft acceptance was dependent on the intragraft infiltration of CD8+ T_CM_ cells (62), suggesting that a regulatory subset of CD8+ T_CM_ cells may be induced in that model. Importantly, it's likely that not all CD44^high^ T cells in our model are alloreactive. Thus, there is a limitation in defining alloreactive CD8+ T_CM_ cells using CD44 marker. To further determine an effect of berberine on donor-specific CD8+ T_CM_ generation, we therefore used an adoptive transfer model of pre-activated Thy1.1+CD8+ T cells with at least two divisions in an MLR *in vitro*, we found that berberine indeed reduced alloreactive Thy1.1+CD8+ T_CM_ cells in recipient mice. It remains to be defined if berberine can also hinder the formation of CD8+ T_CM_ cells in humans.

We validated the alteration of two microbial species, including *Bacteroides ov*. and *Bacillus ce*., by quantitative qPCR. *Bacteroides ov*., a species member of *Bacteroidaceae* family that is a predominant group in gut bacteria of almost all mammals (63), was previously reported to alleviate LPS-induced inflammation in mice (64) and was increased in berberine-treated recipient mice in our study. Nevertheless, we found that colonization of *Bacteroides ov*. alone was insufficient to ameliorate acute allograft rejection. On the other hand, we demonstrated that berberine reduced overall levels of *Bacillus ce*., the toxin-producing bacteria capable of causing human diarrhea, while recolonization of *Bacillus ce*. reversed long-term islet allograft survival induced by berberine plus brief costimulatory blockade, implying that *Bacillus ce*. is a pathogen precipitating allograft rejection. More importantly, our mechanistic studies revealed that colonization of *Bacillus ce*. increased CD8+ T_eff_ and T_CM_ cells whereas addition of *Bacteroides ov*. did not. This finding underscores differences in their immunomodulation and suggests that berberine can eliminate bacterial species that would otherwise promote alloreactive CD8+ T_CM_ generation. Indeed, we found that *Bacillus ce*. contained a cross-reactive antigen that stimulated the formation of alloreactive CD8+ T_CM_ cells, because *Bacillus ce*. rapidly stimulated *in vitro* proliferation and IFN-γ expression by alloreactive CD8+ T_CM_ cells derived from recipient mice that were previously transplanted or primed with specific donor organs. It is possible that endotoxins released by *Bacillus ce*. could also stimulate CD8+ T cells *in vitro*. Fortunately, the response to endotoxins, released possibly by the bacteria, would not be so fast, such as 24 h, because this response was an alloreactive memory recall.

Taken together, berberine promotes the induction of allograft tolerance via downregulating memory CD8^+^ T cells through altering the gut microbiota because: (1) Berberine promoted long-term allograft survival, downregulated CD8+ Tcm and lowered *Bacillus cereus* abundance; (2) Addition of CD8+ Tcm or *B. cereus* reversed allograft survival induced by berberine plus costimulatory blockade; and (3) Furthermore, CD8+ Tcm cells recovered once *B. cereus* was added back to the recipients. Our findings could be implicated for clinical transplantation since berberine has already been widely used to treat gastroenteritis and diarrhea in China (65, 66). Berberine can support a healthy balance of the gut microbiota (67). Furthermore, previous studies have reported that berberine has several pharmacological properties, including improvement of insulin resistance and cholesterol- or glucose-lowering activities (29, 68–72). A recent clinical trial in individuals with ulcerative colitis also showed that berberine reduced the Geboes grade in colonic tissue of patients (73). Thus, berberine can be used to treat multiple diseases in clinic.

## Data Availability Statement

The datasets presented in this study can be found in online repositories. The names of the repository/repositories and accession number(s) can be found at: NCBI Sequence Read Archive, PRJNA693134.

## Ethics Statement

This animal study was reviewed and approved by the Animal Ethics Committee of Guangdong Provincial Academy of Chinese Medical Sciences.

## Author Contributions

FQ performed experiments and wrote the original manuscript. WL provided critical experimental design and some reagents. SY, HL, and QZe performed some experiments. HH, C-LL, and YC worked on some animal experiments. FZ and QZh analyzed the data. C-JL provided main ideas and vital reagents. ZD designed the study and edited the manuscript. All authors contributed to the article and approved the submitted version.

## Conflict of Interest

The authors declare that the research was conducted in the absence of any commercial or financial relationships that could be construed as a potential conflict of interest.

## Abbreviations

ABX: broad-spectrum antibiotics
Ber: berberine
dLN: draining lymph node
EOMES: eomesodermin
Treg: regulatory T cell
T_CM_: central memory T cell
T_EM_: effector memory T cell
T_eff_: effector T cell.
