# Supplementary material for: Berberine Promotes Induction of Immunological Tolerance to an Allograft via Downregulating Memory CD8+ T-Cells Through Altering the Gut Microbiota
Source: Front Immunol. 2021 Feb 12;12:646831. doi: 10.3389/fimmu.2021.646831 (PMC7907598; doi:10.3389/fimmu.2021.646831)

Supplementary data

Figure S1. Berberine has no any effect on CD4+Foxp3+ Treg number

Draining lymph node (dLN) and splenic cells from berberine- or ABX-treated C57BL/6 recipients were isolated 14 days after islet allotransplantation. The frequency of CD4+Foxp3+ Tregs from dLNs and spleens was determined using a flow cytometer, as shown in the density plots (**A**), while the absolute number of the Tregs was also calculated (**B**). Data of individual values are shown as median ± interquartile range (*P<0.05, n = 6-8 mice/group from three separate experiments).

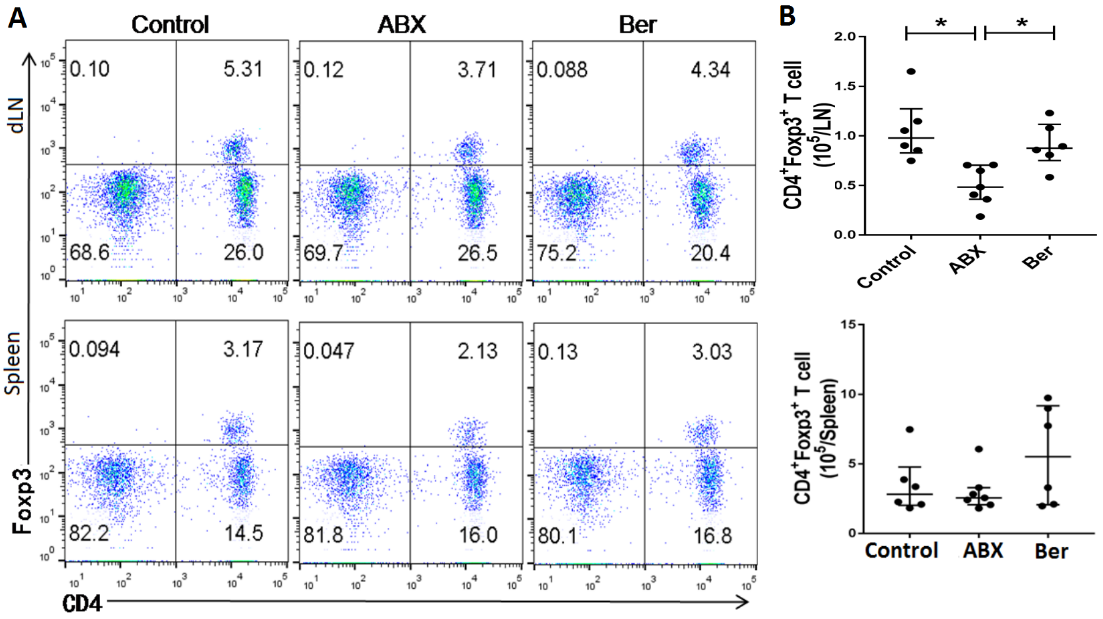

Figure S2. Reduction in "central memory" CD8+ T_{CM} cell number in naïve mice treated with berberine but without transplantation

Spleen and LN cells from naïve B6 mice were isolated 4 weeks after berberine or ABX treatment via oral gavage. The absolute cell number of $CD8^{+}CD44^{high}CD62L^{high}$ (T_{CM}) cells per LN/spleen was measured using FACS analysis. Data are presented as means ± SEM (N = 5-6 mice/group, *p < 0.05). The data showed that berberine significantly reduced CD8+ T_{CM} cell numbers in spleen or LNs 4 weeks after the treatment with berberine, but not ABX.

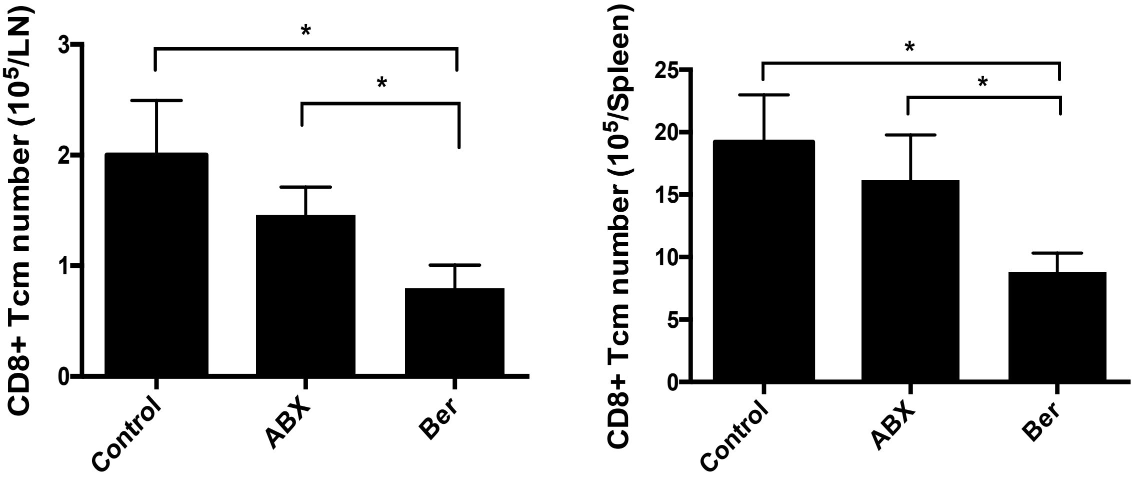

Figure S3. Impacts of berberine on the generation of CD4+/CD8+ T_{CM} cells in recipient mice adoptively transferred with Thy1.1+ naïve T cells

B6 mice were transplanted/primed with BALB/c skin one day after they were adoptively transferred with $2.5x10^6$ $CD4+CD44^{low}$ or $CD8+CD44^{low}$ naïve T cells isolated from Thy1.1+ C57BL/6 mice. Recipient mice were treated with berberine or ABX daily. $CD44^{high}CD62L^{high}$ CD4+/CD8+ T_{CM} cell numbers within Thy1.1+ population were quantified using a flow cytometer four weeks after transplantation. Data are presented as means ± SEM (N = 5-6 mice/group).

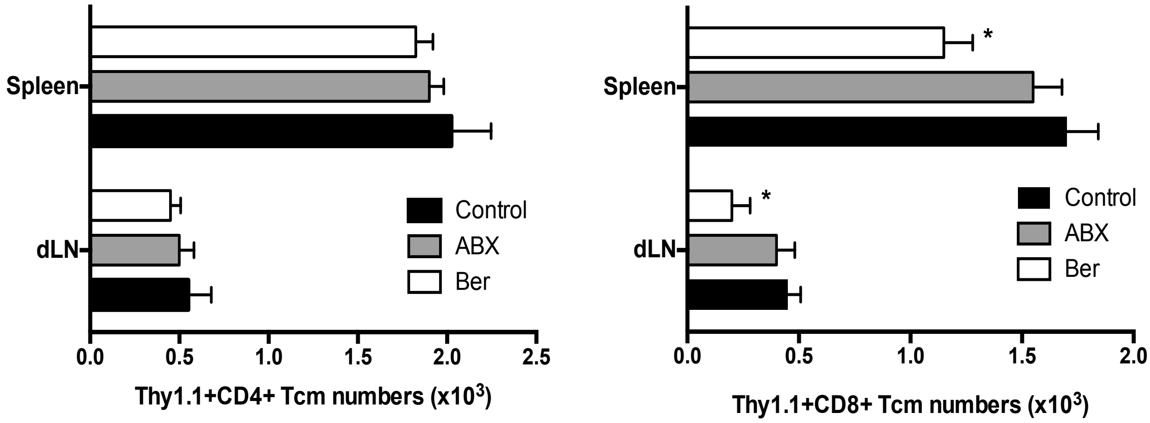

Figure S4. Impact of berberine on alloreactive CD8+ T_{CM} generation in recipient mice adoptively transferred with previously activated Thy1.1+CD8+ T cells

CD3+ T cells isolated from naïve Thy1.1+ B6 mice were stained with CFSE and stimulated with irradiated BALB/c splenocytes in an MLR for 4 days. Thy1.1+CD8+ T cells that underwent at least two divisions were sorted out via FACS sorter, and the pre-activated cells (2.5×10^6) were injected into wild-type B6 mice that were then transplanted with BALB/c islets. Recipient mice were treated orally with berberine or ABX daily for four weeks. $CD44^{high}CD62L^{high}$ CD8+ T_{CM} cell number within Thy1.1+ population was quantified via FACS. Data are presented as means ± SEM (N = 5-6 mice/group, *$p < 0.05$ compared to control). The results showed that alloreactive Thy1.1+CD8+ T_{CM} cell numbers were significantly decreased by the treatment with berberine, but not ABX.

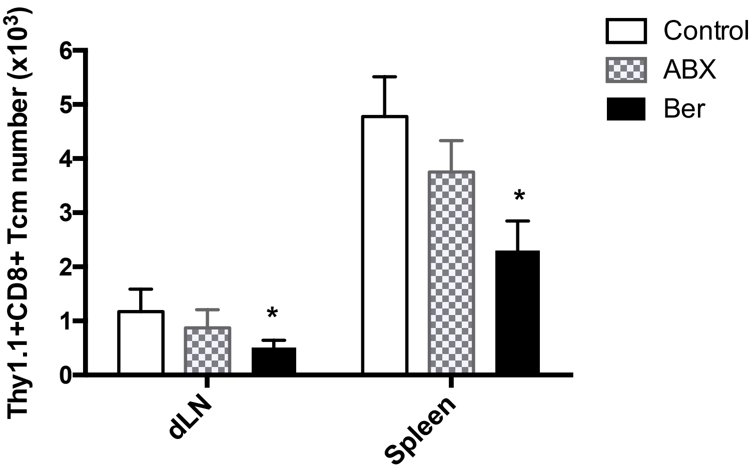

Figure S5. Analysis of cellular apoptosis in recipient mice treated with berberine or ABX

Spleen cells from recipient mice were isolated 14 days after islet transplantation plus either berberine or ABX treatment. The in vivo cytotoxicity of berberine and ABX to cells, including CD4+, $CD8^+$ T, CD19+ and CD11c+ cells, was detected using a flow cytometer and expressed as the percentage of apoptotic cells. Data are presented as means ± SEM (*P<0.05, n = 4 -6 mice/group). Results indicate that berberine is not cytotoxic in this model.

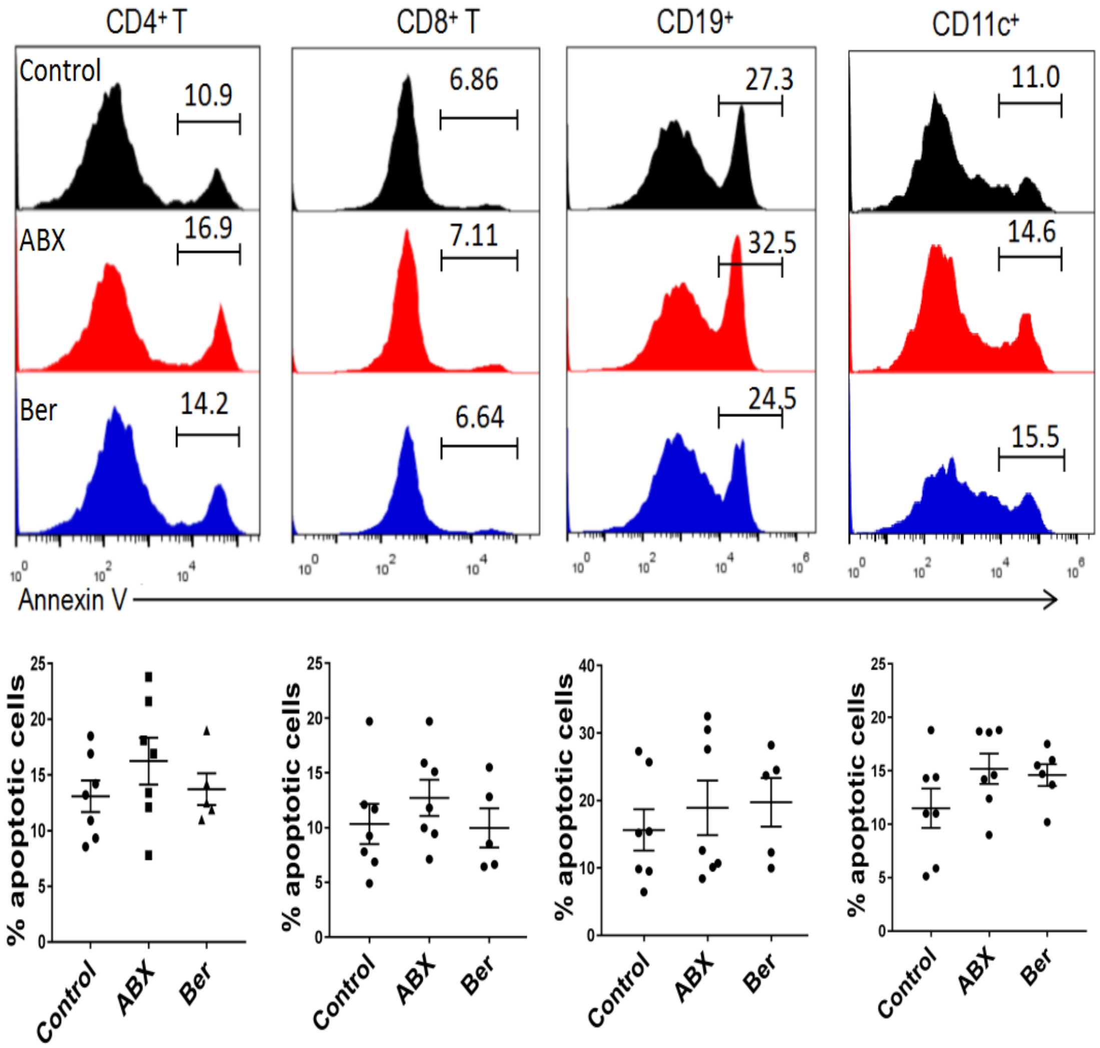

Supplement: Supplementary file 1 [file Data_Sheet_1.PDF]
